# Supplementary material for: Carriers of ADAMTS13 Rare Variants Are at High Risk of Life-Threatening COVID-19
Source: Viruses. 2022 May 29;14(6):1185. doi: 10.3390/v14061185 (PMC9227269; doi:10.3390/v14061185)
Supplement: Supplementary file 1 [file viruses-14-01185-s001.zip › viruses-1668776-supplementary.pdf]

GEN-COVID Multicenter Study (https://sites.google.com/dbm.unisi.it/gen-covid)

Francesca Mari[1,2,3], Mirella Bruttini[1,2,3], Ilaria Meloni[1,2], Susanna Croci[1,2], Gabriella Doddato[1,2], Viola Bianca Serio[1,2], Mirjam Lista[1,2], Debora Maffeo[1,2], Elena Pasquinelli[1,2], Ludovica Mercuri[1,2], Giulia Brunelli[1], Rossella Tita[3], Maria Antonietta Mencarelli[3], Caterina Lo Rizzo[3], Anna Maria Pinto[3], Francesca Ariani[1,2,3], Francesca Montagnani[1,22], Mario Tumbarello[1,22], Ilaria Rancan[1,22], Massimiliano Fabbiani[22], Elena Bargagli[23], Laura Bergantini[23], Miriana D'Alessandro[23], Paolo Cameli[23], David Bennett[23], Federico Anedda[24], Simona Marcantonio[24], Sabino Scolletta[24], Federico Franchi[24], Maria Antonietta Mazzei[25], Susanna Guerrini[25], Edoardo Conticini[26], Luca Cantarini[26], Bruno Frediani[26], Marco Feri[27], Alice Donati[27], Raffaele Scala[28], Luca Guidelli[28], Genni Spargi[29], Marta Corridi[29], Cesira Nencioni[30], Leonardo Croci[30], Gian Piero Caldarelli[31], Davide Romani[32], Paolo Piacentini[32] , Maria Bandini[32], Elena Desanctis[32], Silvia Cappelli[32], Anna Canaccini[33], Agnese Verzuri[33], Valentina Anemoli[33], Manola Pisani[33], Alessandro Pancrazzi[19], Massimo Vaghi[34], Antonella D'Arminio Monforte[35], Federica Gaia Miraglia[35], Mario U. Mondelli[36,37], Stefania Mantovani[36], Raffaele Bruno[36,37], Marco Vecchia[36], Marcello Maffezzoni[38], Sophie Venturelli[10], Andrea Cossarizza[39], Andrea Antinori[40], Alessandra Vergori[40], Arianna Emiliozzi[40], Arianna Gabrieli[6], Agostino Riva[5,6], Andrea Tommasi[7], Pier Giorgio Scotton[41], Francesca Andretta[41], Stefano Baratti[12], Renzo Scaggiante[42], Francesca Gatti[42], Francesco Castelli[43], Eugenia Quiros-Roldan[43], Melania Degli Antoni[43], Isabella Zanella[44,45], Matteo della Monica[13], Roberta Russo[14,15], Immacolata Andolfo[14,15], Achille Iolascon[14,15], Giuseppe Fiorentino[46], Massimo Carella[47], Marco Castori[47], Giuseppe Merla[14,48], Gabriella Maria Squeo[48], Filippo Aucella[49], Pamela Raggi[50], Rita Perna[50], Matteo Bassetti[51,52], Antonio Di Biagio[51,52], Maurizio Sanguinetti[53,54], Luca Masucci[53,54], Alessandra Guarnaccia[53], Serafina Valente[55], Alex Di Florio[55], Marco Mandalà[56], Alessia Giorli[56], Lorenzo Salerni[56], Patrizia Zucchi[57], Pierpaolo Parravicini[57], Elisabetta Menatti[58], Tullio Trotta[59], Ferdinando Giannattasio[59], Gabriella Coiro[59], Fabio Lena[60], Gianluca Lacerenza[60], Cristina Mussini[61], Luisa Tavecchia[62], Lia Crotti[63,64,65,66,67], Gianfranco Parati[63,64], Roberto Menè[63,64], Maurizio Sanarico[68], Marco Gori[69,70], Nicola Picchiotti[69,71], Francesco Raimondi[72], Alessandra Stella[73], Filippo Biscarini[73], Tiziana Bachetti[74], Maria Teresa La Rovere[75], Maurizio Bussotti[76], Serena Ludovisi[77], Katia Capitani[1,78], Chiara Gabbi[79], Simona Dei[80], Sabrina Ravaglia[81], Rosangela Artuso[17], Elena Andreucci[17], Angelica Pagliazzi[17], Erika Fiorentini[17], Antonio Perrella[82], Francesco Bianchi[1,82], Paola Bergomi[83], Emanuele Catena[83], Riccardo Colombo[83], Paola Petrocelli[8], Sarah Iacopini[8], Sara Modica[8], Silvia Baroni[84], Giulia Micheli[85], Marco Falcone[86], Giusy Tiseo[86], Chiara Barbieri[86], Tommaso Matucci[86], Davide Grassi[87], Claudio Ferri[87], Franco Marinangeli[88], Francesco Brancati[89], Antonella Vincenti[90], Valentina Borgo[90], Stefania Lombardi[90], Mirco Lenzi[90], Massimo Antonio Di Pietro[91], Francesca Vichi[91], Benedetta Romanin[91], Letizia Attala[91], Cecilia Costa[91], Andrea Gabbuti[91], Alessio Bellucci[91], Marta Colaneri[92], Patrizia Casprini[93], Cristoforo Pomara[94], Massimiliano Esposito[94], Marco Antonio Bellini[95]

22. Department of Medical Sciences, Infectious and Tropical Diseases Unit, Azienda Ospedaliera Universitaria Senese, Siena, Italy

23. Unit of Respiratory Diseases and Lung Transplantation, Department of Internal and Specialist Medicine, University of Siena, Italy

24. Dept of Emergency and Urgency, Medicine, Surgery and Neurosciences, Unit of Intensive Care Medicine, Siena University Hospital, Italy

25. Department of Medical, Surgical and Neuro Sciences and Radiological Sciences, Unit of Diagnostic Imaging, University of Siena, Italy

26. Rheumatology Unit, Department of Medicine, Surgery and Neurosciences, University of Siena, Policlinico Le Scotte, Italy

27. Dept of Emergency, Anesthesia Unit, San Donato Hospital, Arezzo, Italy

28. Cardiothoraconeurovacular Department, Pneumology and Respiratory intensive Care a unit, San Donato Hospital, Arezzo, Italy

29. Department of Emergency, Anesthesia Unit, Misericordia Hospital, Grosseto, Italy

30. Department of Specialized and Internal Medicine, Infectious Diseases Unit, Misericordia Hospital, Grosseto, Italy

31. Clinical Chemical Analysis Laboratory, Misericordia Hospital, Grosseto, Italy

32. Dipartimento di Prevenzione, Azienda USL Toscana Sud Est, Italy

33. Dipartimento Tecnico-Scientifico Territoriale, Azienda USL Toscana Sud Est, Italy

34. Chirurgia Vascolare, Ospedale Maggiore di Crema, Italy

35. Department of Health Sciences, Clinic of Infectious Diseases, ASST Santi Paolo e Carlo, University of Milan, Italy

36. Division of Clinical Immunology - Infectious Diseases, Department of Medicine, Fondazione IRCCS Policlinico San Matteo, Pavia, Italy

37. Department of Internal Medicine and Therapeutics, University of Pavia, Italy

38. University of Pavia, Pavia, Italy

39. Department of Medical and Surgical Sciences for Children and Adults, University of Modena and Reggio Emilia, Modena, Italy

40. HIV/AIDS Department, National Institute for Infectious Diseases, IRCCS, Lazzaro Spallanzani, Rome, Italy

41. Department of Infectious Diseases, Treviso Hospital, Local Health Unit 2 Marca Trevigiana, Treviso, Italy

42. Infectious Diseases Clinic, ULSS1, Belluno, Italy

43. Department of Infectious and Tropical Diseases, University of Brescia and ASST Spedali Civili Hospital, Brescia, Italy

44. Department of Molecular and Translational Medicine, University of Brescia, Italy;

45. Clinical Chemistry Laboratory, Cytogenetics and Molecular Genetics Section, Diagnostic Department, ASST Spedali Civili di Brescia, Italy

46. Unit of Respiratory Physiopathology, AORN dei Colli, Monaldi Hospital, Naples, Italy

47. Division of Medical Genetics, Fondazione IRCCS Casa Sollievo della Sofferenza Hospital, San Giovanni Rotondo, Italy

48. Laboratory of Regulatory and Functional Genomics, Fondazione IRCCS Casa Sollievo della Sofferenza, San Giovanni Rotondo, Italy

49. Department of Medical Sciences, Fondazione IRCCS Casa Sollievo della Sofferenza Hospital, San Giovanni Rotondo, Italy

50. Clinical Trial Office, Fondazione IRCCS Casa Sollievo della Sofferenza Hospital, San Giovanni Rotondo, Italy
51. Department of Health Sciences, University of Genova, Genova, Italy
52. Infectious Diseases Clinic, Policlinico San Martino Hospital, IRCCS for Cancer Research Genova, Italy
53. Microbiology, Fondazione Policlinico Universitario Agostino Gemelli IRCCS, Catholic University of Medicine, Rome, Italy
54. Department of Laboratory Sciences and Infectious Diseases, Fondazione Policlinico Universitario A. Gemelli IRCCS, Rome, Italy
55. Department of Cardiovascular Diseases, University of Siena, Siena, Italy
56. Otolaryngology Unit, University of Siena, Italy
57. Department of Internal Medicine, ASST Valtellina e Alto Lario, Sondrio, Italy
58. Study Coordinator Oncologia Medica e Ufficio Flussi, Sondrio, Italy
59. First Aid Department, Luigi Curto Hospital, Polla, Salerno, Italy
60. Department of Pharmaceutical Medicine, Misericordia Hospital, Grosseto, Italy.
61. Infectious Diseases Clinics, University of Modena and Reggio Emilia, Modena, Italy
62. U.O.C. Medicina, ASST Nord Milano, Ospedale Bassini, Cinisello Balsamo (MI), Italy
63. Istituto Auxologico Italiano, IRCCS, Department of Cardiovascular, Neural and Metabolic Sciences, San Luca Hospital, Milan, Italy
64. Department of Medicine and Surgery, University of Milano-Bicocca, Milan, Italy
65. Istituto Auxologico Italiano, IRCCS, Center for Cardiac Arrhythmias of Genetic Origin, Milan, Italy
66. Istituto Auxologico Italiano, IRCCS, Laboratory of Cardiovascular Genetics, Milan, Italy
67. Member of the European Reference Network for Rare, Low Prevalence and Complex Diseases of the Heart-ERN GUARD-Heart
68. Independent Data Scientist, Milan, Italy
69. University of Siena, DIISM-SAILAB, Siena, Italy
70. Maasai, I3S CNRS, Université Côte d’Azur, France
71. Department of Mathematics, University of Pavia, Pavia, Italy
72. Laboratorio di Biologia Bio@SNS, Scuola Normale Superiore, Pisa, Italy
73. CNR-Consiglio Nazionale delle Ricerche, Istituto di Biologia e Biotecnologia Agraria (IBBA), Milano, Italy
74. Direzione Scientifica, Istituti Clinici Scientifici Maugeri IRCCS, Pavia, Italy
75. Istituti Clinici Scientifici Maugeri IRCCS, Department of Cardiology, Institute of Montescano, Pavia, Italy
76. Istituti Clinici Scientifici Maugeri IRCCS, Department of Cardiology, Institute of Milan, Italy
77. Fondazione IRCCS Ca’ Granda Ospedale Maggiore Policlinico, Milan, Italy
78. Core Research Laboratory, ISPRO, Florence, Italy
79. Department of Biosciences and Nutrition, Karolinska Institutet, Stockholm, Sweden
80. Health Management, Azienda USL Toscana Sud Est, Tuscany, Italy

81. IRCCS C. Mondino Foundation, Pavia, Italy

82. Department of Medicine, Pneumology Unit, Misericordia Hospital, Grosseto, Italy.

83. Department of Anesthesia and Intensive Care Unit, ASST Fatebenefratelli Sacco, Luigi Sacco Hospital, Polo Universitario, University of Milan, Milan

84. Department of Diagnostic and Laboratory Medicine, Unity of Chemistry, Biochemistry and Clinical Molecular Biology, Fondazione Policlinico Universitario A. Gemelli IRCCS, Catholic University of the Sacred Heart, Rome, Italy.

85. Clinic of Infectious Diseases, Catholic University of the Sacred Heart, Rome, Italy

86. Department of Clinical and Experimental Medicine, Infectious Diseases Unit, University of Pisa, Pisa, Italy

87. Department of Clinical Medicine, Public Health, Life and Environment Sciences, University of L'Aquila, Italy

88. Anesthesiology and Intensive Care, University of L'Aquila, L'Aquila, Italy

89. Medical Genetics Unit, Department of Life, Health and Environmental Sciences, University of L'Aquila, L'Aquila, Italy

90. Infectious Disease Unit, Hospital of Massa, Italy

91. Infectious Diseases Unit, Santa Maria Annunziata Hospital, USL Centro, Florence, Italy

92. Division of Infectious Diseases I, Fondazione IRCCS Policlinico San Matteo, Pavia, Italy

93. Laboratory of Clinical Pathology and Immunoallergy, Florence-Prato, Italy

94. Department of Medical, Surgical and Advanced Technologies "G.F. Ingrassia", University of Catania, Catania, Italy

95. Ambulatory Chronic Polipathology of Siena, Department of Medicine, Surgery and Neurosciences, University of Siena, Siena, Italy
